# An mHealth Intervention to Improve Guardians’ Adherence to Children’s Follow-Up Care for Acute Lymphoblastic Leukemia in Tanzania (GuardiansCan Project): Protocol for a Development and Feasibility Study

**DOI:** 10.2196/48799

**Published:** 2023-08-02

**Authors:** Faraja S Chiwanga, Joanne Woodford, Golden M Masika, David A Richards, Victor Savi, Louise von Essen

**Affiliations:** 1 Department of Women's and Children's health Healthcare Sciences and e-Health Uppsala University Uppsala Sweden; 2 Directorate of Medical Services Muhimbili National Hospital Dar es Salaam United Republic of Tanzania; 3 Department of Clinical Nursing University of Dodoma Dodoma United Republic of Tanzania; 4 Department of Health and Caring Sciences Western Norway University of Applied Sciences Bergen Norway

**Keywords:** childhood cancer, eHealth, feasibility, guardians, intervention development, low- and middle-income countries, Tanzania, mHealth, mobile health, public contribution, public involvement, mHealth intervention, leukemia, psychological distress

## Abstract

**Background:**

Cancer is a leading cause of death during childhood and in low- and middle-income countries survival rates can be as low as 20%. A leading reason for low childhood cancer survival rates in low- and middle-income countries such as Tanzania is treatment abandonment. Contributing factors include poor communication between health care providers and children’s guardians, insufficient cancer knowledge, and psychological distress.

**Objective:**

Our aim is to respond to Tanzanian guardians’ poor adherence to children’s follow-up care after treatment for acute lymphoblastic leukemia with the help of mobile health (mHealth) technology. Our goal is to increase guardians’ adherence to children’s medications and follow-up visits and to decrease their psychological distress.

**Methods:**

Following the Medical Research Council framework for developing and evaluating complex interventions, we will undertake the GuardiansCan project in an iterative phased approach to develop an mHealth intervention for subsequent testing. Public contribution activities will be implemented throughout via the establishment of a Guardians Advisory Board consisting of guardians of children with acute lymphoblastic leukemia. We will examine the acceptability, feasibility, and perceived impact of Guardians Advisory Board activities via an impact log and semistructured interviews (study I). In phase 1 (intervention development) we will explore guardians’ needs and preferences for the provision of follow-up care reminders, information, and emotional support using focus group discussions and photovoice (study II). We will then co-design the mHealth intervention with guardians, health care professionals, and technology experts using participatory action research (study III). In phase 2 (feasibility), we will examine clinical, methodological, and procedural uncertainties associated with the intervention and study procedures to prepare for the design and conduct of a future definitive randomized controlled trial using a single-arm pre-post mixed methods feasibility study (study IV).

**Results:**

Data collection for the GuardiansCan project is anticipated to take 3 years. We plan to commence study I by recruiting Guardians Advisory Board members in the autumn of 2023.

**Conclusions:**

By systematically following the intervention development and feasibility phases of the Medical Research Council Framework, and working alongside an advisory board of guardians, we intend to develop an acceptable, culturally appropriate, feasible, and relevant mHealth intervention with the potential to increase guardians’ adherence to children’s follow-up care after treatment of acute lymphoblastic leukemia, leading to a positive impact on children’s health and chances to survive, and reducing distress for guardians.

**International Registered Report Identifier (IRRID):**

PRR1-10.2196/48799

## Introduction

### Overview

Cancer is a leading cause of death during childhood [1,2]. In high-income countries over 80% of children are cured [3], whereas in low- and middle-income countries (LMIC) survival rates can be as low as 20% [4]. If childhood cancer treatment inequalities are not addressed, it is estimated that 11.1 million children will not survive childhood cancer between 2020 and 2050, with 9.3 million (84%) of these cases living in LMIC [5]. Avoidable deaths from childhood cancer in LMIC result from factors including lack of diagnosis, misdiagnosis or delayed diagnosis, financial barriers, lack of essential chemotherapy, drug toxicity, poor survivorship care, reliance on traditional and complementary medicine, and treatment abandonment [4-8]. Deficiencies in pediatric cancer care are particularly pronounced within sub-Saharan Africa [9].

A major reason for low survival rates in LMIC is treatment abandonment, which is defined as failure to start (refusal) or continue curative treatment for 4 or more consecutive weeks [10-12]. Commonly reported factors contributing to treatment abandonment include dissatisfaction with health care providers’ communication, guardians’ lack of knowledge about cancer, preferences for traditional and complementary medicine, and psychological distress alongside limited access to psychological support [13,14]. In a Kenyan study, parents of children receiving treatment for leukemia reported anxiety, shock, fatigue, and economic challenges, which were associated with heightened family tension and increased need for emotional support [15]. Our research in Tanzania has identified that guardians report poor communication between themselves and health care providers, insufficient cancer knowledge, and emotional concerns [16], with findings suggesting that guardians have unmet needs related to health provider communication, information about childhood cancer, and emotional support. Importantly, these unmet needs can have negative consequences for guardians’ adherence to the child’s follow-up care, for example, medication adherence and follow-up appointments, which can have an impact on the child’s health and chances of survival. However, research from Malawi suggests receiving information about treatment duration, reasons for and frequency of medical procedures, and the positive outcome in many cases of common childhood cancers can reduce treatment abandonment [17].

Mobile health (mHealth) interventions may represent a solution to address guardians’ unmet needs, reduce treatment abandonment, and ultimately improve childhood cancer survival rates. Specifically, mHealth interventions may provide guardians with follow-up treatment reminders, educational information about cancer and treatment, and emotional support. Across sub-Saharan Africa, national-level digital health strategies and architectures are being implemented and are endorsed by the United Nations and the World Health Organization [18]. mHealth interventions have shown promise in improving treatment adherence among patients with tuberculosis in Tanzania [19] and young people living with HIV in sub-Saharan Africa [20]. A recent systematic review of the use of 2-way SMS text message interventions to improve appointment attendance and medicine adherence in sub-Saharan Africa found improvements in medicine adherence in comparison to standard care, but not appointment attendance. However, effectiveness differed between clinical conditions [21]. While an mHealth intervention represents a promising solution to improve adherence to the child’s follow-up care, more knowledge is needed regarding the relationship between different types of mHealth interventions (eg, apps or SMS text messaging) and different outcomes including appointment attendance and medicine adherence. In addition, although mHealth technology has been piloted in many sub-Saharan African countries for various clinical and public health interventions, most of these interventions have not been scaled up due to poor design, limited stakeholder involvement, and neglecting to consider the implementation context [22].

mHealth interventions could also provide emotional support to guardians. mHealth apps using cognitive behavioral therapy (CBT) principles are effective for common mental health difficulties [23], and we have successfully used internet-administered CBT to improve depression and anxiety in Swedish parents of children during [24,25] and after [26] cancer treatment. An mHealth intervention informed by CBT principles that supports guardians’ unmet emotional needs may facilitate their adherence to the child’s treatment and follow-up care. In addition to the uncertainties regarding which type of mHealth intervention should be used, and how to integrate CBT principles to target emotional needs, mHealth interventions should also be tailored to the target population [19]. Tailoring includes careful consideration of the population’s needs and preferences, attitudes toward mHealth interventions, and perceived barriers and facilitators to intervention use. Potential barriers related to the technology, the health care system, and community factors, such as households sharing mobile phones, should also be carefully considered [27]. In addition, the acceptability and feasibility of the intervention and trial methods should be explored before undertaking a definitive randomized controlled trial (RCT) [28]. This paper describes the design of the GuardiansCan project in which we aim to respond to Tanzanian guardians’ poor adherence to children’s follow-up care after treatment for acute lymphoblastic leukemia (ALL) with the help of mHealth technology.

### Overall Aim and Specific Goals

Our goal is to increase guardians’ adherence to children’s medications and follow-up visits and, second, to decrease their psychological distress. Our overall objectives are to (1) implement public contribution activities throughout the GuardiansCan project via the establishment of a Guardians Advisory Board (GAB) and to examine the acceptability, feasibility, and perceived impact of GAB activities (study I); (2) to explore guardians’ needs and preferences concerning the provision of follow-up care reminders, information, and emotional support (study II); (3) to co-design an mHealth intervention providing guardians with follow-up care reminders, relevant information, and emotional support (study III); and (4) to examine clinical, methodological, and procedural uncertainties associated with the intervention and study procedures to prepare for the design and conduct of a future definitive RCT of the mHealth intervention (study IV).

## Methods

### Overview

Following the revised United Kingdom Medical Research Council (MRC) framework for developing and evaluating complex interventions [29], we will undertake the GuardiansCan project in an iterative phased approach to develop an mHealth intervention for subsequent testing. The GAB will contribute to the project [30-32] and follow a study-focused framework [33] with public contribution activities planned at the managing and undertaking, analysis and interpretation, and dissemination phases of the project. As GAB members will be involved in designing the detailed research protocols for studies II-IV, we provide here an overview of the methods and study procedures as planned for now, to be further informed via GAB contribution.

**Figure 1:**
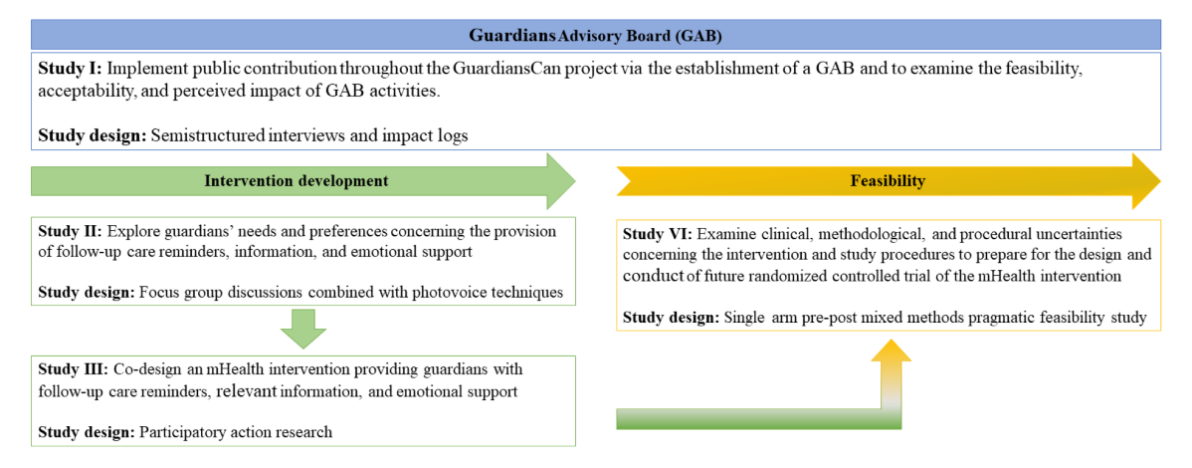
Overview of the GuardiansCan project. mHealth: mobile health.

The GuardiansCan project is divided into two main phases in accordance with the MRC Framework: (1) intervention development and (2) feasibility, with data anticipated to be collected over a 3-year period. We will follow published guidance to conduct the intervention development phase, paying attention to involving stakeholders, reviewing published evidence, developing a program theory, and considering future real-world implementation [29,34], and the feasibility phase regarding the design and reporting of feasibility trials [35] and supplementary guidelines for nonrandomized feasibility studies [36]. An overview of the GuardiansCan project can be seen in Figure 1.

### Setting

The GuardiansCan project is an international collaboration between Tanzania and Sweden. Uppsala University (Sweden) is the responsible academic institution with research primarily coordinated from Muhimbili National Hospital (MNH), a National Referral and University Teaching Hospital located in Dar es Salaam, Tanzania. MNH provides basic and advanced medical and surgical treatments, and is a strategic center for the implementation of eHealth aligning with the Tanzania National Road map of eHealth Investment 2017-2023 [37]. MNH is the main hospital for the treatment of pediatric cancer in Tanzania and the center for the pediatric oncology network that established a pediatric oncology unit at MNH in 2013 with collaborative support from the government, nongovernmental organizations, and other donors. The pediatric oncology unit includes “Upendo children’s cancer ward” and the “Ujasiri house,” a hostel for families. Around 100 children are diagnosed annually with ALL at the unit.

Regarding general access to mobile phones, Tanzania’s mobile subscription rate per 100 population is 85%, with Android (Google) being the dominant operating system [38,39]. A study among guardians of children with cancer showed that 85% had mobile phones; however, out of these, only 9% reported having smartphones [40].

### Recruitment

**Figure 2:**
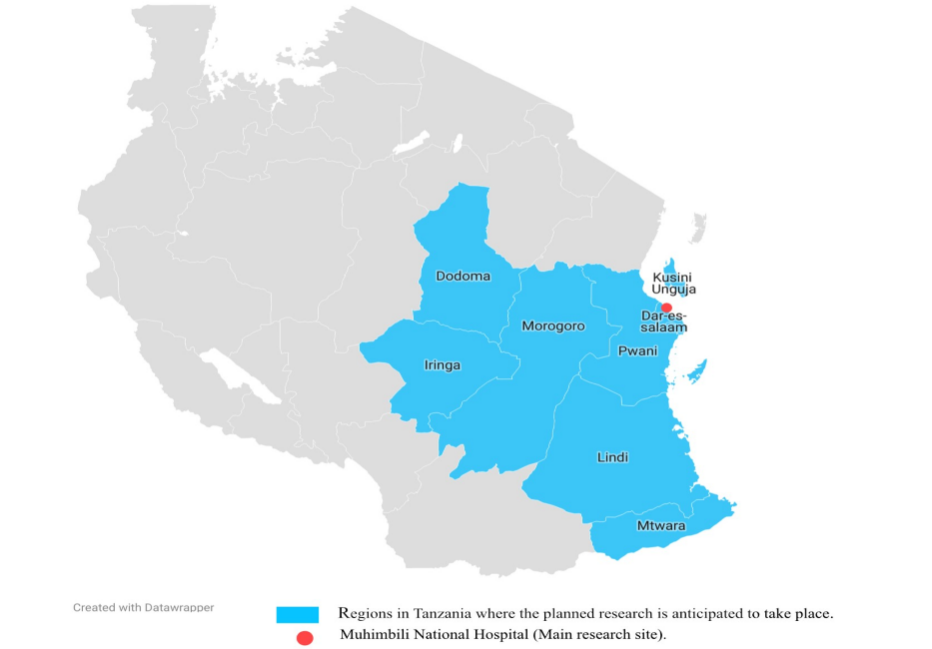
Map of regions in Tanzania where the planned research is anticipated to take place.

For each planned study, we intend to recruit guardians from 7 regions in Tanzania including Dar es Salaam, Dodoma, Iringa, Lindi, Morogoro, Mtwara, and Pwani, and the island of Zanzibar (a semiautonomous state and part of the United Republic of Tanzania). The regions represent urban and rural areas in Tanzania and have been selected to facilitate including guardians with different cultural backgrounds and accessibility to Dar es Salaam (see Figure 2). Recruitment will be conducted in collaboration with pediatric oncologists or pediatric oncology nurses at the pediatric oncology unit at MNH. Oncologists or nurses will identify potential participants for each study via MNH records. An oncologist or a nurse will call identified guardians to explore inclusion criteria and their initial interest in each study. Subsequently, a member of the research team will call interested guardians to provide more study information, undertake screening procedures, and obtain informed consent. The detailed recruitment strategy for study I will be published elsewhere. An overview of planned recruitment procedures is presented for studies II-IV as planned for now; however, recruitment strategies are subject to change via collaboration with the GAB.

### Public Contribution in Research

#### Guardians Advisory Board: Study I

##### Aims

We aim to involve guardians of children treated for ALL in the managing and undertaking, analysis and interpretation, and dissemination phases of the GuardiansCan project, and examine the acceptability, feasibility, and perceived impact of GAB members’ contribution to the GuardiansCan project from the perspective of GAB members and public contribution coordinators.

##### Methods

The full public contribution plan for the GuardiansCan project will be published elsewhere. We aim to recruit 6 to 8 guardians of children treated for ALL at MNH, representing different genders and coming from rural and urban communities, into the GAB. Workshops will be held with GAB members, facilitated by public contribution coordinators who are Kiswahili speakers with GAB members contributing to the undertaking and managing, analysis and interpretation, and dissemination phases of the GuardiansCan project. Examples of public contribution activities include, but are not limited to, designing detailed study protocols for studies II-IV, assisting in writing participant information sheets and consent forms, data collection (eg, cofacilitating or observing focus groups), data interpretation and sense-making of findings, developing a dissemination plan, and helping to distribute findings within community settings.

##### Data Collection

Public contribution activities will be recorded in an impact log [41] immediately after each GAB workshop. Workshop discussions will be held in Kiswahili and will be audio-recorded; however, audio-recordings will not be transcribed and only made to ensure impact log accuracy. Semistructured interviews will also be conducted to explore the acceptability, feasibility, and perceived impact of GAB activities from the perspective of GAB members and public contribution coordinators 6 months after GAB has been formed and at the end of studies II-IV. Semistructured interviews will be conducted in Kiswahili and audio-recorded.

##### Data Analysis

Researchers in our group who are not involved in public contribution activities and conversant with Kiswahili will read impact logs, with impacts (eg, anticipated and unanticipated changes to the research process and potential benefits and harms) extracted and summarized. Summaries of impacts will be presented for discussion with GAB members and public contribution coordinators to aid sense-making and interpretation of results. Two members of the research team who are not involved in GAB activities will analyze semistructured interviews using manifest content analysis [42]. Descriptions of subcategories and categories will be presented for discussion with GAB members and public contribution coordinators to aid in sense-making and interpretation of results.

### Phase 1: Intervention Development

#### Intervention Program Theory

Following the MRC Framework [29] throughout the intervention development phase we will use findings iteratively to develop and refine an intervention program theory [34]. The program theory will describe: (1) how we expect the mHealth intervention to increase guardians’ adherence to children’s medications and follow-up visits; (2) pathways predicted to drive the change; and (3) under what conditions the change is expected to occur [43]. The program theory will be presented diagrammatically, in the form of a logic model. Cumulative information from the intervention development phase will be used to identify intervention program inputs, activities, outputs, and the context in which they operate to deliver short- and long-term outcomes of interest [44]. We will continue to iteratively build the program theory and logic model throughout intervention development and later test and refine the developed program theory during the feasibility phase [34].

#### Focus Group Discussion and Photovoice: Study II

##### Aims

We aim to explore (1) perceived needs and preferences concerning the receipt of follow-up care reminders, information, and emotional support; (2) attitudes toward mHealth interventions; and (3) perceived barriers and facilitators of mHealth intervention use.

##### Study Design

An explorative qualitative design with data collected via focus group discussions combined with photovoice techniques will be used [45-47]. Photovoice is a qualitative research method whereby participants are engaged actively in the research process, using photographs or pictures to facilitate discussion and generate knowledge around a particular research topic, with an overall aim to reach policy makers to generate societal change concerning an important community issue [47,48].

##### Inclusion Criteria

Participants will be guardians of the children treated for ALL at MNH and health care professionals (eg, nurse practitioners, pediatric oncologists, pediatric oncology nurses, pediatricians, pharmacists, and social workers) working at the pediatric oncology unit at MNH. We will include guardians who meet the following inclusion criteria: (1) are at least 18 years of age; (2) whose child has been discharged within the last 3-36 months from MNH after receiving main treatment for ALL (ie, completion of the induction phase of treatment); (3) are able to speak Kiswahili; (4) have completed primary school education (International Standard Classification of Education [ISCED] level 1 to postsecondary nontertiary education ISCED level 4), but not tertiary education; (5) can provide written informed consent via ink signature; and (6) are able to travel to attend focus group discussions.

##### Recruitment

A member of the research team will speak to interested health care professionals working at the pediatric oncology unit at MNH face-to-face or over the telephone to provide more study information. Those who wish to participate will be provided with a study information sheet and consent form. Written informed consent or alternatively verbal recorded consent over the telephone or via a secure videoconferencing system will be obtained. After provision of informed consent, background and sociodemographic characteristics will be collected, that is, age, gender, profession, length of time in profession, professional qualifications, and length of time working with pediatric oncology.

An oncologist or a nurse will call identified guardians to explore inclusion criteria and their initial interest. A member of the research team will call guardians who fulfill the inclusion criteria and are interested in participation to provide more detailed verbal study information. Guardians interested in participating will be invited to the first focus group discussion. We will provide guardians with (1) a study information sheet and (2) consent form and written informed consent (via ink signature) will be obtained prior to data collection.

##### Data Collection

A series of 2 focus group discussions with health care professionals (n≈6-8) at MNH will be held in Kiswahili. We will hold a series of 6 focus group discussions combined with photovoice with guardians (n≈3-4 females and n≈3-4 males). We plan to conduct focus groups in Dar es Salaam at a venue where participants can share their thoughts and feelings in privacy. It is anticipated that guardians will meet approximately once every 2 weeks for a 12-week period with each focus group lasting approximately 3 hours. However, the exact venue, frequency, and duration of focus group discussions will be planned in collaboration with the GAB.

Focus groups with guardians will be combined with photovoice techniques [45-47]. We will predevelop topics for each focus group in collaboration with GAB members and they will be conducted in Kiswahili. During the first focus group with guardians, we will present study aims and participants will be trained in photovoice procedures, how to use a camera, and in ethical considerations [49]. Next, we will introduce the topic for the subsequent focus group discussion and participants will take 3 photographs based on the topic prior to the subsequent planned focus group. During each of the focus group discussions, photographs will be used to elicit discussions around each preplanned topic. At the end of each focus group, the topic for the subsequent focus group will be introduced.

Focus group discussions will be moderated and notes taken with the assistance of an observer, a GAB member who will help facilitate discussions. We will audio-record focus group discussions and they will be transcribed verbatim by a person conversant in Kiswahili. An external translation company will translate transcripts to English and back-translate it to Kiswahili to facilitate analysis by the whole research team. Transcripts will be anonymized.

##### Data Analysis

Data will be analyzed using manifest content analysis [42]. Two members of the research team will individually code transcripts and perform categorization of codes into categories and subcategories. Subsequently, categories and subcategories will be discussed in the wider research team to facilitate interpretation. Descriptions of subcategories and categories will be presented for discussion with GAB members to aid sense-making and interpretation of results. Visual data from photographs will be used as supplementary data sources. Trustworthiness of the analysis will be established in accordance with a trustworthiness checklist for content analysis [50] and by adopting procedures including an audit trail, case analysis for deviant cases, and peer examination.

#### Participatory Action Research: Study III

##### Aims

We aim to identify: (1) intervention content informed by findings from study II, behavioral change techniques [51-53] and evidence-based CBT principles [23,54], and may include audio, visual, and written elements; (2) acceptable methods of intervention delivery within the health care context (eg, 1-way SMS text messaging, automatic voice message reminders, 2-way SMS text messaging, or via artificial intelligence methods, or a basic or smartphone app) which are suitable for urban and rural environments; (3) intervention delivery preferences, for example, technical elements [55,56] such as reminders and personalization and whether a professional group is needed to support intervention delivery; and (4) potential technical support and training needs of guardians and health care professionals.

##### Study Design

A qualitative design based on principles from participatory action research (PAR) will be used [57,58], placing key stakeholders at the center of the research process.

##### Inclusion Criteria

Participants include guardians of the children treated for ALL at MNH, meeting the same inclusion criteria as study II, and health care professionals working in pediatric oncology care, and technology experts, including mobile operator technicians.

##### Recruitment

We anticipate identifying health care professionals and technology experts via in-person networks and guardians via MNH patient records, following the same recruitment procedures used in study II.

##### Data Collection

Approximately 8 face-to-face PAR iterative meetings will be held in Kiswahili at a venue where participants can share their thoughts and feelings in privacy. Each meeting is anticipated to last approximately 3 hours, including a refreshment break. Before the first meeting, background and sociodemographic characteristics from participants will be collected. Some meetings will only include guardians or health care professionals and technology experts, and some meetings will be combined.

Each meeting will have a predefined theme to focus the discussions and we will predevelop topics in collaboration with GAB members. PAR meetings will be conducted in iterative cycles and each meeting will involve cycles of collaboration, discussion, reflection, and evaluation. At the beginning of each meeting, we will present the agenda and participants will be encouraged to add items to the agenda. Action plans related to the topic will be developed collaboratively and at the end of each meeting, participants will individually reflect on the group discussions by answering open-ended questions and will be encouraged to provide suggestions for the upcoming meeting [58]. An initial intervention prototype will be drafted after 6 meetings. We will discuss the initial prototype in combined meetings and come up with the proposed intervention to be tested in the feasibility phase.

PAR meetings will be facilitated and notes taken with the assistance of an observer, who will be a GAB member. Meetings will be audio-recorded and will be transcribed verbatim by a person native in Kiswahili. An external translation company will translate transcripts to English and back-translate to Kiswahili to facilitate analysis by the whole research group. Transcripts will be anonymized. Participants’ input at meetings and on action plans will be discussed by meeting facilitators, the research team, and the GAB who jointly plan further actions.

##### Data Analysis

Data will be analyzed (meeting notes, action plans, and transcripts) using manifest content analysis [42] in a “recursive” process to reach a collaborative understanding of the data. Two members of the research team including a Tanzanian national will individually code transcripts and perform the categorization of codes into categories and subcategories. Subsequently, categories and subcategories will be discussed in the wider research team to facilitate interpretation. The analysis will be presented for discussion with GAB members to aid sense-making and interpretation of results. Trustworthiness of the analysis will be established in accordance with a trustworthiness checklist for content analysis [50] and by adopting procedures including an audit trail, case analysis for deviant cases, and peer examination. We will develop a full prototype of the mHealth intervention describing the type of intervention, information to be provided, and mode and frequency of delivery for feasibility testing.

### Phase 2: Feasibility

#### Single-Arm Pre-Post Mixed Methods Pragmatic Feasibility Study: Study IV

##### Aims

We aim to determine (1) intervention acceptability from the perspectives of guardians and health care professionals; (2) feasibility of the intervention; and (3) feasibility and acceptability of study methods and procedures.

##### Study Design

We will undertake a single-arm pre-post mixed methods pragmatic feasibility study [35,36,59].

##### Inclusion Criteria

Participants will be guardians (1 per child) whose child has been discharged from MNH after receiving main treatment for ALL (ie, completion of the induction phase of treatment) with the inclusion or exclusion criteria informed by studies II and III findings and GAB member involvement.

##### Recruitment

Exact recruitment procedures will be informed by GAB member involvement. It is anticipated the recruitment period will take 6 months. To examine recruitment feasibility, the study invitation materials will include the possibility for guardians who decline participation to provide reasons for nonparticipation and consent to take part in an interview to explore potential decisions not to participate. Written informed consent (via ink signature) will be obtained prior to data collection and being provided with access to the intervention. In addition to the intervention, guardians will receive care from health care professionals as per normal clinical practice.

We anticipate recruiting a sample of between 20 and 40 participants, following recommendations for feasibility studies [60] and, as in other single-arm pragmatic feasibility studies [61], to provide information on feasibility outcomes and inform the sample size for a future definitive RCT [62].

##### Data Collection

We plan to collect data at screen, baseline, and posttreatment follow-up (12 months post intervention). In line with our feasibility objectives [35], we will collect the following outcomes: (1) intervention acceptability from the perspectives of guardians and health care professionals (eg, intervention acceptability, intervention usability, and indications for usage such as number of opened messages); (2) feasibility of delivering the intervention (eg, number of successfully delivered messages); and (3) feasibility and acceptability of study methods and procedures (eg, number of guardians eligible and enrolled, data collection completeness, and feasibility of data collection procedures). Progression criteria and stopping rules will be defined [63] to inform progression to an RCT. The exact progression criterion and stopping rules will be developed in accordance with our feasibility objectives, existing relevant literature, and input from a broad range of relevant stakeholders, including GAB members and researchers external to the research team who have relevant expertise in conducting similar research [64].

To examine the feasibility of data collection, the following clinical data will be collected: (1) adherence to child’s medications as per discharge instructions (eg, amount of medication taken and successful prescription refill); (2) attendance of follow-up visits at MNH or another hospital as per discharge instructions; (3) number and types of complications after discharge, number of readmissions after discharge, and progress of children’s cancer; and (4) psychological distress as measured with the Kiswahili adaptation of the Kessler Psychological Distress scale [65], cancer knowledge, and quality of life [66]. We will examine the feasibility of collecting economic data to inform a future cost-effectiveness evaluation, for example, provider costs pertaining to direct financial costs of delivering and maintaining the intervention and personnel costs [67]. Semistructured interviews at posttreatment will be conducted in Kiswahili to explore intervention acceptability, relevance, impact (eg, on medication adherence, attendance at outpatient follow-up visits, guardians’ psychological distress, cancer knowledge, and child’s well-being), and barriers and facilitators to intervention use. All guardians included in the study will be invited to participate in the interviews until data saturation is met. We will also interview health care professionals involved in the intervention to explore experiences of the intervention and perceived impact on guardians.

##### Data Analysis

Participant flow will be illustrated in accordance with the CONSORT (Consolidated Standards of Reporting Trials) diagram for feasibility and pilot studies [35]. We will calculate the percentages of participants (1) consenting, (2) screened, (3) meeting eligibility criteria, and (4) enrolled calculated with 95% CIs. Reasons for ineligibility, ambiguities regarding eligibility criteria, and reasons for nonparticipation at each stage will be reported. We will calculate follow-up rates, and the numbers of missing items relating to outcome measures are calculated with 95% CIs. We will report descriptive statistics (means and SDs or proportions with 95% CIs) for each clinical outcome at eligibility screen, baseline, and posttreatment (eg, adherence to child’s medications, attendance of follow-up visits, number and types of complications and readmissions, psychological distress). We will also report frequencies of intervention activity relating to intervention adherence and use. We will analyze semistructured interviews using manifest content analysis [42] and establish the trustworthiness of the analysis in accordance with a trustworthiness checklist for content analysis [50] and by adopting procedures including an audit trail, case analysis for deviant cases, and peer examination. Quantitative and qualitative data will be analyzed separately, with findings integrated following a triangulation protocol at the participant level to explore potential relationships between mHealth intervention implementation, intervention acceptability and perceived impact, and changes in adherence to medication and follow-up visits and psychological distress. Joint displays will be used to compare quantitative and qualitative findings [68,69]. We will present the analysis for discussion with GAB members to aid sense-making and interpretation of results.

### Reporting

We will report: (1) GAB activities and perceived impact of activities following the GRIPP2 (Guidance for Reporting Involvement of Patients and the Public) checklist [70]; (2) qualitative results in accordance with the SRQR (Standards for Reporting Qualitative Research) checklist [71]; (3) intervention development in line with GUIDED (Guidance for Reporting Intervention Development Studies in Health Research) [44]; (4) intervention content following the TIDieR (Template for Intervention Description and Replication) checklist [72] and the mERA (mHealth Evidence Reporting and Assessment) checklist [73]; and (5) feasibility study results in accordance with the CONSORT extension for reporting randomized pilot and feasibility [35].

### Data Management

We will process data in accordance with the Swedish Patient Data Act (2008:355), the General Data Protection Regulation (EU 2016/679), and the Tanzania National Institute of Medical Research policy [74]. Tanzanian and Swedish members of the research group will have access to collected data and will be involved in data management and analysis. We will develop a detailed data management plan for each study that will be made available on DMPonline. In summary, research data will be pseudonymized and a list linking ID number and identifiable personal information (ie, participant name and contact details) will be kept separately from research data. Data will be stored on password-protected USB sticks kept in a locked fireproof cabinet at MNH and stored in ALLVIS, a secure web-based data portal used by Uppsala University for the confidential storage of research data, accessible by both Tanzanian and Swedish members of the research group. A Data Transfer Agreement will be completed between Uppsala University and MNH.

### Ethical Considerations

The GuardiansCan project will be conducted in accordance with the Declaration of Helsinki [75]. Informed consent (via ink signature) will be obtained from all GAB members and study participants. We will obtain ethics approval for all studies from the Swedish Ethical Review Authority, MNH Ethics Review Board, and the Tanzania National Research Ethics Committee; and research permits for foreign researchers will be sought from the Tanzania Commission for Science and Technology for study IV. Given the iterative nature of the planned research and involvement of the GAB, ethics approval will be obtained for all 4 studies in a stepwise manner, with ethics approvals initially obtained for study I.

Members of the GAB (study I) will receive reimbursement for their contribution, which includes travel and living cost and an allowance equivalent to the government daily subsistence allowance for ISCED level 4 personnel: 75,000 Tanzanian Shillings (≈US $31). Guardians participating in study II will receive mobile phones to facilitate the use of photovoice techniques. Guardians participating in studies II-IV will also receive reimbursement for any travel and living costs incurred.

## Results

Data collection for the GuardiansCan project is anticipated to take 3 years. We plan to commence study I by recruiting Guardians Advisory Board members in the autumn of 2023.

## Discussion

### Principal Findings

The development of interventions to address childhood cancer treatment inequalities and avoidable deaths from childhood cancer in LMIC is a high public health priority. To the best of our knowledge, this is the first study to seek to increase guardians’ adherence to children’s medications and follow-up visits after treatment for ALL, decrease guardians’ psychological distress via the use of mHealth, and represents an opportunity to increase childhood cancer survival rates in LMIC such as Tanzania.

Despite the piloting of mHealth interventions across sub-Saharan African countries, mHealth interventions are seldom scaled-up due to factors such as lack of stakeholder involvement and consideration of the implementation context [22]. To overcome these limitations, robust methods will be adopted systematically following the intervention development and feasibility phases of the revised MRC complex interventions research framework [29], developing program theory, and involving wider stakeholder groups throughout the intervention development phases. In addition, we will implement public contribution throughout the GuardiansCan project by working alongside an advisory board of guardians with lived experience to enhance the quality and relevance of the GuardiansCan project, ensuring local applicability and acceptance, and a feasible and sustainable solution to a significant health care challenge. Consequently, important data will be generated to facilitate the development of an acceptable, culturally appropriate, feasible, and relevant intervention, as well as maximize the success of a future definitive RCT. Significant stakeholder involvement and consideration of context and future real-world implementation will enhance the future implementation potential of the intervention should it later be demonstrated to be clinically and cost-effective. In addition, we will use our developed program theory to inform future process evaluations of the intervention.

### Limitations

We did not involve guardians as public contributors when developing the overall research plan, limiting our ability to plan study procedures in advance that maximize acceptability and feasibility for guardians. However, we plan to overcome this limitation by embedding public contribution via the GAB throughout the GuardiansCan project, including activities related to the managing and undertaking, analysis and interpretation, and dissemination phases of the project. As planned currently, we will only recruit guardians from 7 geographical regions of Tanzania. Therefore, results may not be generalizable to guardians located in other regions of Tanzania, including guardians located at very long distances from the pediatric oncology unit at MNH. We will not include guardians who have not completed at least primary school; therefore, the proposed mHealth intervention may not be suitable for guardians who have not attained primary education.

### Conclusions

The proposed mHealth intervention and research have significant potential to promote equitable and sustainable care for families struck by ALL in Tanzania, adding to the capacity building of health care services in Tanzania. Our research may have a positive impact on children’s health and chances to survive, and guardians’ ability to provide for the family as a result of reduced costs for treatments, transportation, and long periods of hospitalization. The mHealth intervention also has significant potential to be a clinical and cost-effective solution, optimally prepared alongside key stakeholders for the implementation and a real-world clinical impact. The project, and the novel and robust methodological approach being taken, will also add to the existing evidence base concerning how mHealth interventions should be developed and tested, which may be of benefit to patients and informal caregivers for a variety of different conditions and unmet needs.

## Abbreviations

ALL: acute lymphoblastic leukemia
CBT: cognitive behavioral therapy
CONSORT: Consolidated Standards of Reporting Trials
GAB: Guardian Advisory Board
GRIPP2: Guidance for Reporting Involvement of Patients and the Public
GUIDED: Guidance for Reporting Intervention Development Studies in Health Research
ISCED: International Standard Classification of Education
LMIC: low- and middle-income countries
mERA: mHealth Evidence Reporting and Assessment
mHealth: mobile health
MNH: Muhimbili National Hospital
MRC: Medical Research Council
PAR: participatory action research
RCT: randomized controlled trial
SRQR: Standards for Reporting Qualitative Research
TIDieR: Template for Intervention Description and Replication
